# Fecal egg counts for gastrointestinal nematodes are associated with a polymorphism in the MHC-DRB1 gene in the Iranian Ghezel sheep breed

**DOI:** 10.3389/fgene.2015.00105

**Published:** 2015-03-24

**Authors:** Rahman Hajializadeh Valilou, Seyed A. Rafat, David R. Notter, Djalil Shojda, Gholamali Moghaddam, Ahmad Nematollahi

**Affiliations:** ^1^Department of Animal Science, Faculty of Agriculture, University of Tabriz, TabrizIran; ^2^Department of Animal and Poultry Sciences, Virginia Tech, Blacksburg, VAUSA; ^3^Department of Pathobiology, College of Veterinary Medicine, University of Tabriz, TabrizIran

**Keywords:** nematodes, genetic resistance, FAMACHA, MHC-DRB1, PCR-RFLP, sheep

## Abstract

Genetic variation among sheep breeds in resistance to gastrointestinal nematodes (GIN) has been demonstrated in several production environments. Relationships between the ovine major histocompatibility complex and resistance to GIN have been studied, but few studies have systematically examined this issue in less-developed and semi-arid regions. The aim of the current study was to explore associations between fecal worm egg counts (FEC) for several GIN and polymorphisms in the DRB1 gene. One hundred male lambs were selected at 4–6 months of age from weaned animals in five flocks (*n* = 20 per flock). Body weights were determined, FAMACHA scores based on color of the ocular mucous membranes were assigned as an indicator of anemia, and blood and fecal samples were collected twice to evaluate FEC and blood packed cell volume (PCV) and for DNA isolation. A repeated-measures analysis of variance was used to test effects of genotype on FEC. The model included fixed effects of flock, genotype, time of measurement (1 or 2), and flock × time and genoype × time interactions, and a random (repeated) effect of lamb. Two genotypes (A1A1 and A1A2) were observed following digestion of Region 1 of Ovar-DRB1 with PstI. Genotypic frequencies were 0.73 for A1A1 and 0.27 for A1A2. FEC differed between Ovar_DRB1 genotypes A1A1 and A1A2 for *Marshallagia marshalli*, Strongyle, and total nematode FEC. Observed FEC were 30–41% lower for genotype A1A1. Differences among genotypes were consistent across measurement times, with no effect of genotype × measurement time interaction for any parasite class (*P* ≥ 0.34). A significant association was observed between FAMACHA scores and lamb PCV, and the residual correlation between these two variables was -0.51 (*P* < 0.001). FAMACHA scores can thus be used to detect differences among lambs in PCV, and polymorphic markers of Ovar-DRB1 have potential value as an indicator of parasite resistance in applied animal breeding programs on sheep farms in this region.

## Introduction

The Ghezel sheep is one of the 27 mainly fat-tailed native breeds of Northwestern Iran (Tavakolian, 2000). Animals of this breed graze for much of the year and are therefore continuously exposed to natural nematode infections. Gastrointestinal nematodes (GIN) of sheep and goats are widespread, diverse, and highly pathogenic, and can also infect other ruminant species such as cattle and reindeer (Jacquiet et al., 1998; Achi et al., 2003; Hrabok et al., 2006). Effects of GIN are most extreme in young animals, and therefore represent a real threat to the sheep industry (Waller, 2006).

The development of multi-drug resistance by GIN has driven research into alternative control measures, including selection of sheep that are genetically resistant to GIN infection. Genetic variation among breeds in resistance to GIN has been demonstrated in a variety of production environments. Genetically resistant sheep (either representing resistant local breeds or developed by selection within commercial breeds) are increasingly being used to improve animal production and well-being (Amarante et al., 2009). Genetically resistance sheep types also provide an opportunity to study novel mechanisms of resistance that may not be present in susceptible commercial breeds (Piedrafita et al., 2010). However, to date the mechanisms underlying genetic resistance of sheep to GIN infections are not precisely known.

Evidence for host genetic variation in aspects of disease resistance has now been documented for many diseases and in all major domestic livestock species (Bishop, 2005). In particular, small ruminants are notable for the large number of diseases where host genetic variation has been documented. Because parasite resistance in sheep has a moderate heritability (0.2–0.6; Baker, 1998; Stear et al., 2007), selective breeding has been used successfully with several breeds of sheep in different climates (Gray, 1987). Most sheep breeding programs for GIN resistance are based on recording of faecal egg counts (FEC), but this type of phenotype measurements is costly and difficult to collect on a large scale. In these situations, use of molecular genetic information is an interesting option. Use of molecular markers of resistance to GIN in sheep breeding programs has shown some promise, but difficulties remain, mainly because effects or previously identified quantitative trait loci (QTL) have not been consistent across breeds (Matika et al., 2011).

In sheep, class II genes of the major histocompatibility complex (MHC) are located on chromosome 20 and encode polymorphic glycoproteins composed of nine covalently linked subunits. Gruszczynska et al. (2000) found significant effect of OLA–DRB1 (MHC class II) on body weight at birth of Polish Heath sheep. There is also a body of scientific literature linking genes in the sheep MHC with the ability of sheep to resist infection by GIN as measured by FEC (Schwaiger et al., 1995; Buitkamp et al., 1996; Stear et al., 1996; Paterson et al., 1998). These findings are perhaps not surprising given the role of these genes in controlling specific immune responses. This “MHC effect” is never-the-less thought to be relatively small, accounting for an estimated 11% of total phenotypic variation in traits associated with GIN resistance (Buitkamp et al., 1996), although it accounts for a somewhat larger proportion of the additive genetic variation (Stear et al., 1997). These results have led to speculation that the MHC contains genes that could be used as markers for breeding to reduce FEC but that do not fully explain genetic resistance to GIN.

Few studies have addressed the relationships between the ovine MHC and resistance to GIN in less-developed and semi-arid regions. The current study is part of a multi-national collaborative project sponsored by the International Atomic Energy Agency and the Food and Agricultural Organization of the United Nations and designed to study genetic control of resistance to GIN in local sheep breeds. A particular focus of the study is the abomasal GIN *Haemonchus contortus* and was justified by this parasite’s ability to produce large numbers of eggs, resulting in extensive pasture contamination; the blood-sucking nature of this nematode, which can causes life-threatening levels of anemia; and the associated potential for very significant reductions in lamb performance and survival (Gatongi et al., 1998; Waller et al., 2004). In Iran, Ashrafi et al. (2014) demonstrated polymorphism in exon 2 of MHC gene OLA-DRB1 in the Makui sheep breed of Northwestern Iran. In this study, our aim was thus to explore possible associations between nematode resistance and polymorphism of DRB1. Because *H. contortus* is only one of several GIN known to be present in the temperate regions of Iran, we likewise focused our study on a variety of different GIN known to be important in the region (Garedaghi and Bahavarnia, 2013; Moradpour et al., 2013; Yagoob et al., 2013).

## Material and Methods

### Study Area

**FIGURE 1:**
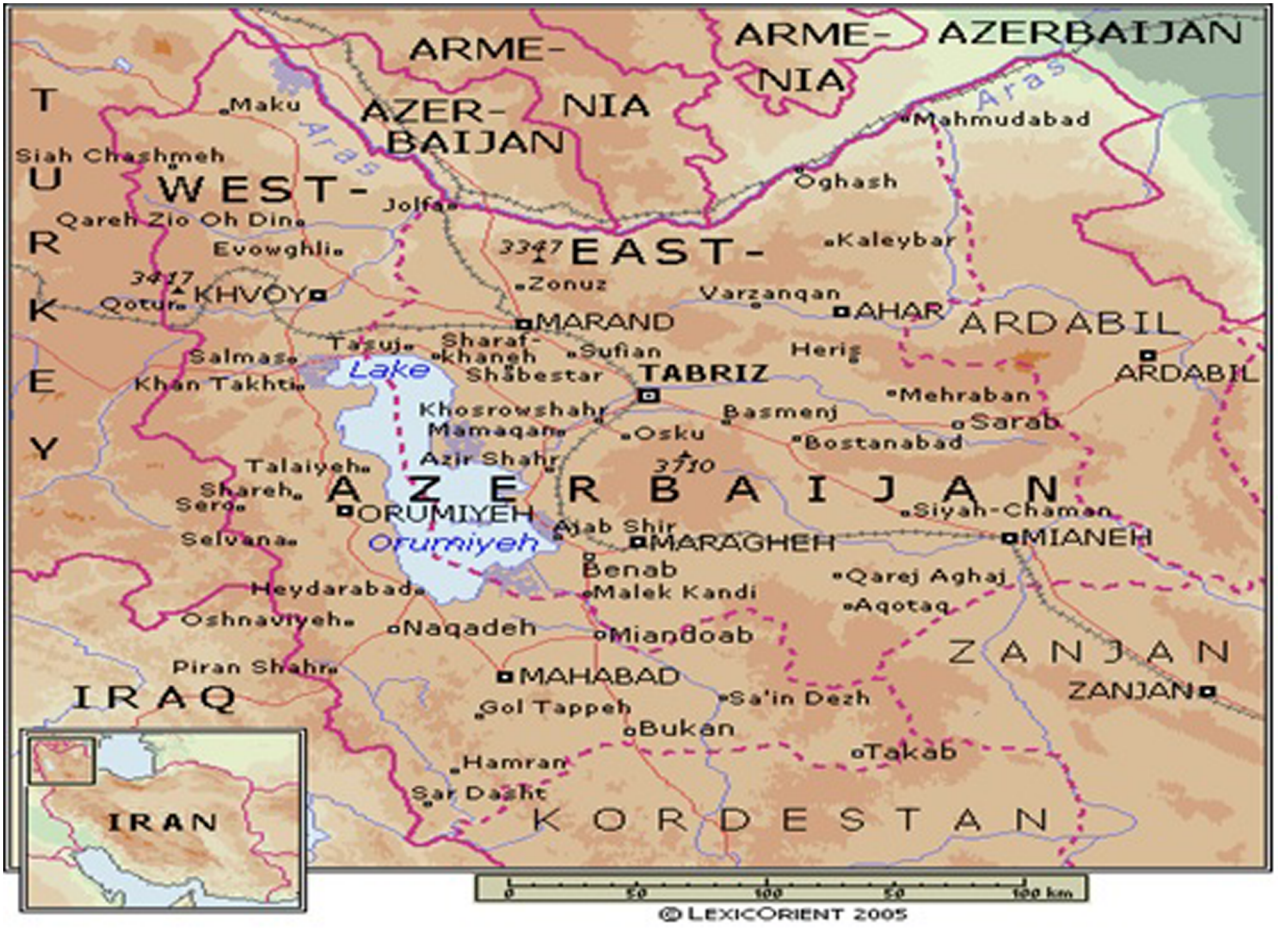
**The study region was located in Northwestern Iran between 35 and 39°North latitude and between 46 and 48°East longitude**.

The study area is shown in **Figure 1**. The districts of Eastern and Western Azerbaijan provinces are agro-ecological zones and these zones are the site of origin and the habitat of the Ghezel sheep breed in Iran (Tavakolian, 2000). This breed was therefore selected for the present study. The study region is located at latitude 35–38.8°North and longitude 46–48°East and receives annual rainfall of 150–350 mm. The temperature is highest in June, before the onset of the monsoon season. During late spring and early to mid-summer, the daily maximum temperature rarely declines below 22°C. Relative humidity is lowest during April and May and rises during the monsoon season. The year is commonly divided into four seasons: winter (December–February), spring (March–April), summer (May–September), and autumn (October–November). The summer also includes the monsoon season (July–August; www.irimo.ir). This study was conducted in May and June, a time when GIN numbers were anticipated to be elevated.

### Animals and Scheduling of Phenotype Sampling

One hundred male lambs at an age of 4–6 months were selected for this study. Each lamb was randomly selected from weaned animals within five flocks (*n* = 20 per flock). After deworming to eliminate existing nematode infection and when a parasite-free condition was confirmed (28 days later), the 20 lambs in each flock were allowed to graze together with untreated contemporaries from the same flock for at least 28 days without deworming. From day 31 post-infection, body weights were determined and blood and fecal samples were collected twice, 1 week apart, to evaluate fecal parasite egg counts and blood packed cell volume (PCV) and for DNA isolation. A single observer assigned FAMACHA scores for all flocks and both sampling times using a scale of 1–5. Scores were based on the color of the ocular mucous membranes surrounding the eye using procedures and color charts described by Vatta et al. (2001). All experimental procedures were approved by the University of Tabriz Animal Care and Ethics Committee.

### Sample Processing

Individual fecal samples were collected from the rectum, processed to determine FEC using the modified McMaster technique, and reported as eggs per gram of feces. Observed parasite ova in the feces were categorized by parent species as: (1) Strongyles, (2) *Nematodirus* sp., (3) *Trichuris* sp., and (4) *Marshallagia marshalli*. The Strongyle group potentially included a number of common abomasal and intestinal sheep nematodes typical of mixed nematode infections in small ruminants such as *H. contortus*, *Teladorsagia circumcincta*, *Ostertagia occidentalis*, and *Trichostrongylus axei*, *colubriformis*, *vitrinus*, and* rugatus*. Fecal egg counts (FEC) were also summed across the four parasite classes for each lamb to derive a total nematode egg count. FEC were determined by the Clayton Lane technique.

Blood was obtained from the jugular vein with sterile vacuum tubes with anticoagulant (EDTA). For each sample, the PCV (%) was determined on the day of collection using the micro-hematocrit method. Blood was then mixed with 0.5 M of EDTA (pH = 8), and frozen at -20°C. DNA was isolated from blood using the protocol of Samadi Shams et al. (2011). Sequences of forward and reverse primers for amplification of the Ovar MHC-DRB1 (Region 2) gene are shown in **Table 1** (Amills et al., 1995).

**Table 1:** Characteristics of PCR primers, nucleotide substitutions, restriction enzymes, amino acid changes, PCR producte size, and digested sequence sizes (allele) sizes for RFLP polymorphisms in Exon 2 of the Ovar-DRB1 gene.

Characteristic	Palindrome 2	Palindrome 2
Primer sequence	5-TAT CCC GTC TCT GCA GCA CAT TTC-3	
	5-TCG CCG CTG CAC ACT GAA ACT CTC-3	
Nucleotide substitution	(CTGCAG to GTGCAG or TTGCAG)	(TCGA to TACG)
Restriction enzyme	PstI	TaqI
Amino acid change	Phe and Tyr to Val and Cys	Phe to Tyr
PCR product(bp)	285	285
Allele size(bp)	(+/+): 241 and 44	(+/+): 163 and 122

The PCR was performed in a 25 μl reaction using the master mix kit (Ampliqon Company) in a T-Personal thermo-cycler (BiometeraPersonal Cycler Version 3.26 co., Germany). The PCR mixture contained: 50–100 ng of DNA, 2.5 μl of 10X PCR buffer (200 mM (NH4)2SO4, 0.1 mM Tween 20%, 750 mM Tris-HCl (pH 8.8), 2.5 mM MgCl2, 200 μM dNTPs, and 3 μl mix of oligonucleotids (10 pmol from each primer), 1U Taq DNA polymerase (Dream Taq polymerase, Ampliqon company) and 11 μl ddH2O. A total of 35 cycles was adapted for denaturation at 94°C/1 min, annealing at 61°C /1 min and polymerization at 72°C/2 min (**Table 2**). The PCR products were electrophoresed at 85 V for 45 min in 2.5% agarose gels, and visualized under UV light. The power supply for electrophoresis was a PAC1000 (Bio-Rad company; USA). The size of the alleles was determined based on a 100 bp DNA size standard (Ampliqon Company) using the computer software BIO 1D++. The PCR product for each sample was digested with 10 units of PstI and TaqI enzymes at 37 and 65°C, respectively. The characterization of each has been given in **Table 3**. The digested products were separated in a 2 and 3% agaros gel for 1 h at 85 V. The gels were stained with ethidium bromide.

**Table 2:** PCR temperatures program.

PCR step	Temperature (C^0^)	Time	Cycles
Elementary denaturation	95	4 min	1
Secondary denaturation	95	1 min	35
Annealing	61	50 s	
Extension	72	1 min	
Termination	72	5 min	1

**Table 3:** Characteristics and features of TaqI and PstI enzymes and restriction sites.

Enzyme	Origination	Unit	Concentration	Restriction site	Optimal performance temperature (C^0^)	Incubation time
PstI	*Providencia stuarti*	3000	10 U/μL	5^′^…C▼TGCAG…3^′^ 3^′^…G ACG▲TT…5 ^′^	37	1–16 h
TaqI	*Thermusa quaticus* YT-1	3000	10 U/μL	5^′^…T▼CG A…3^′^ 3^′^…A GC▲T…5^′^	65	1–16 h

### Statistical Analysis

Fecal egg counts were analyzed separately for each class of parasite and for the total nematode egg count. For each parasite class, FEC values were expressed as residual deviations from flock × time subclass means, and distributions of residuals was tested for skewness (ω) and kurtosis (κ). If FEC were not normally distributed, FEC values were transformed before further analysis using Box–Cox transformations of the form [(FEC – 1)/; Box and Cox, 1964]. Optimum values of for a range of values between -2 and 2 were determined using a maximum-likelihood criterion (Draper and Smith, 1981) in the TransReg Procedure of SAS.

A repeated-measures analysis of variance was conducted using the MIXED Procedure of SAS to test effects of genotype on transformed FEC, body weights, and PCV. The model included fixed effects of flock, genotype, time of measurement (1 or 2), and flock × time and genoype × time interactions and a random (repeated) effect of lamb. This model was also fitted to untransformed FEC using the GLIMMIX Procedure of SAS and assuming a negative binomial distribution of FEC (O’Hara and Kotze, 2010). Associations between FAMACHA scores and measured variables were evaluated by adding effects of FAMACHA score and associated two-way interactions with other fixed effects to this mixed model.

## Results

**FIGURE 2:**
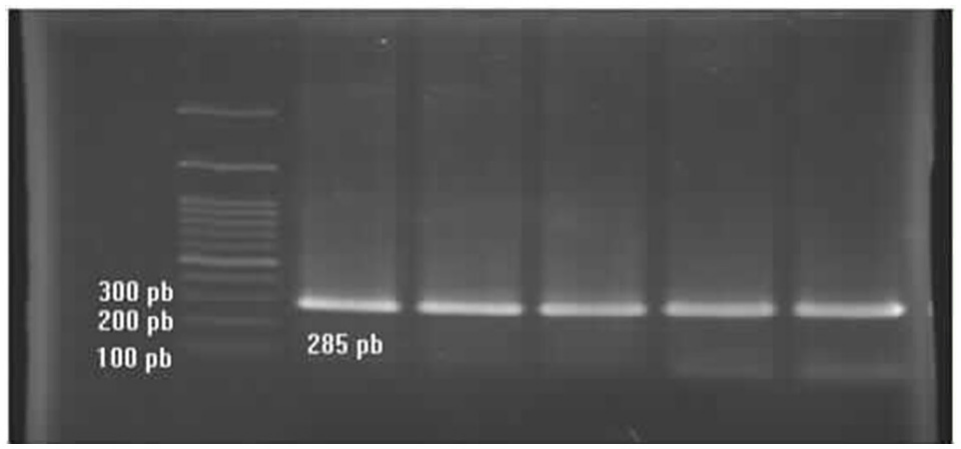
**PCR products of the Ovar-DRB1 gene**.

**FIGURE 3:**
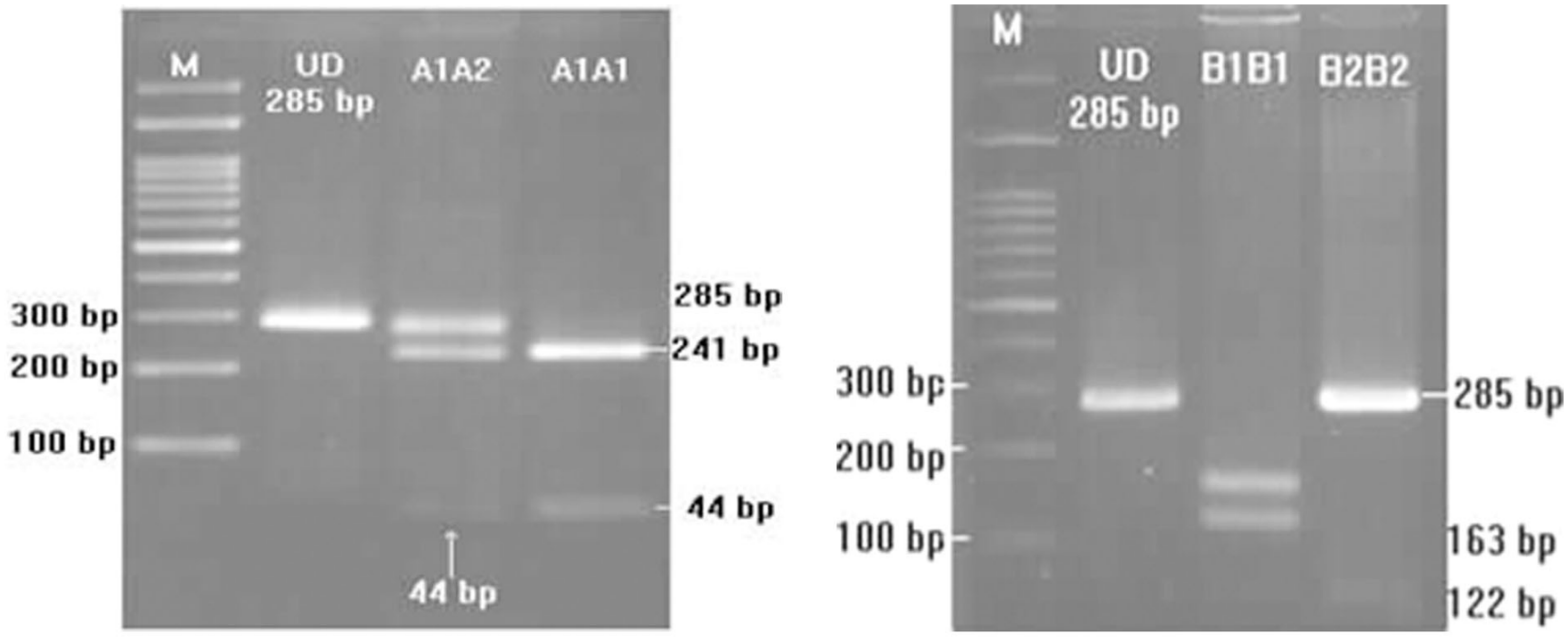
**Genotypes and PCR-RFLP pattern of the digested Ovar-DRB1 gene digested (**Left**: digested with PstI; **Right**: digested with TaqI)**.

**FIGURE 4:**
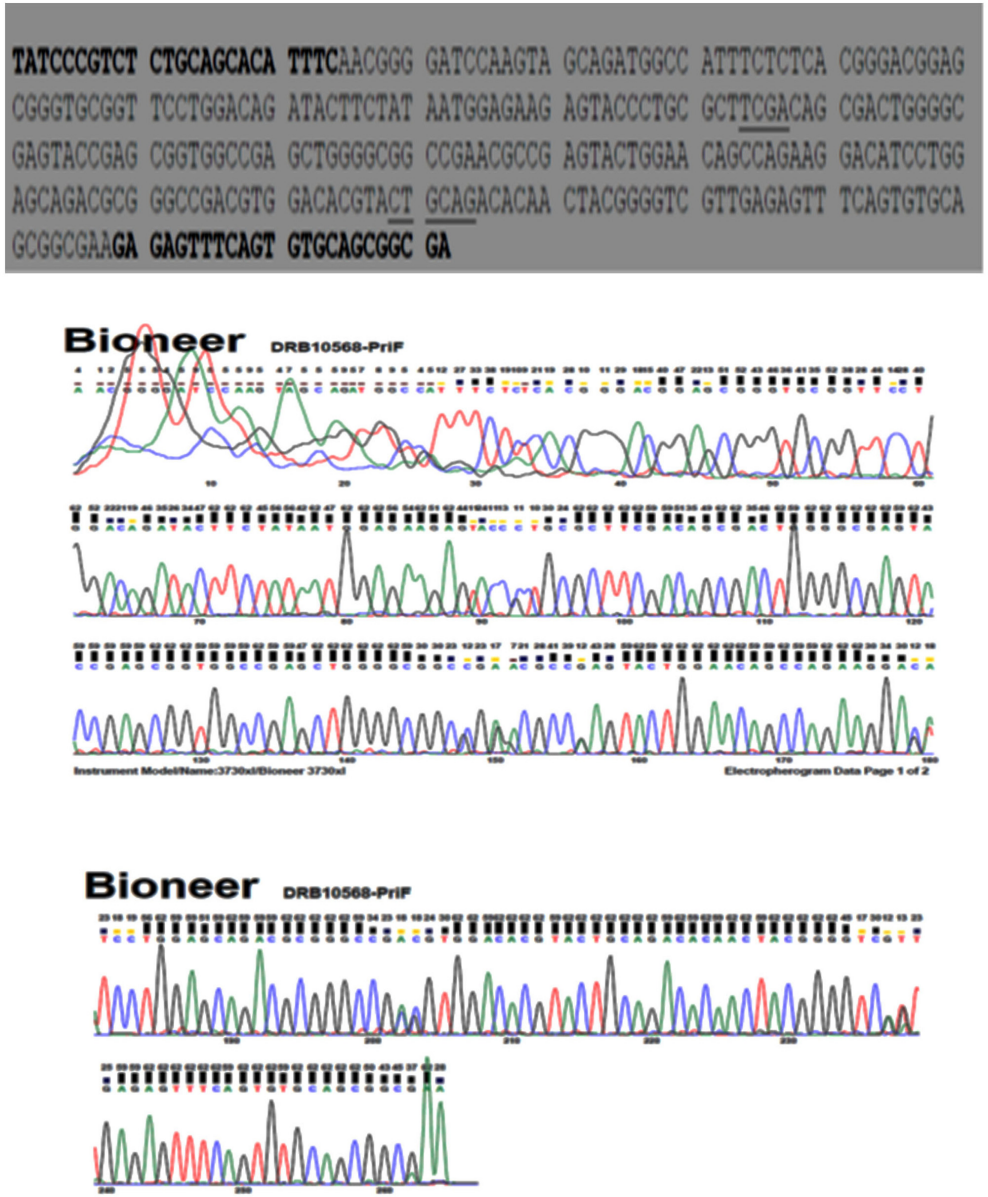
**Nucleotide sequence of the DRB1 allele in one of the samples (animal no 10568).** Primer complementary regions are indicated in bold type while the PstI sites are underlined.

Two genotypes (A1A1 and A1A2) were observed following digestion of Region 1 of Ovar-DRB1 with PstI (**Figures 2** and **3**). Two genotypes (B1B1 and B2B2) were likewise obtained following digestion with TaqI, but only one lamb had genotype B2B2 and effects of the TaqI polymorphism were therefore not considered further. For the PstI polymorphism, both genotypes were present in each of the five flocks, with overall genotypic frequencies of 0.73 for A1A1 and 0.27 for A1A2 (**Table 4**). The observed number of alleles at these two loci was similar to that pervious reported by Amills et al. (1995). The PCR product was sequenced (*n* = 4; Bioneer, Munpyeongseo-ro, Daedeok-gu, Daejeon 306–220, Republic of Korea). **Figure 4** illustrates the sequence.

**Table 4:** Genotype and gene frequencies.

Genotypes^1^	Numbers of lambs					Overall genotypic frequencies
	Flock 1	Flock 2	Flock 3	Flock 4	Flock 5	
A1A1	15	10	16	17	15	0.73
A1A2	5	10	4	3	5	0.27

^1^Alleles A1 and A2 were identified following digestion of Ovar-DRB1 with PstI.

Alleles B1 and B2 were identified following digestion with TaqI (**Figure 3**).

Corresponding overall allelic frequencies were 0.865 and 0.135 for A1 and A2, respectively.

**FIGURE 5:**
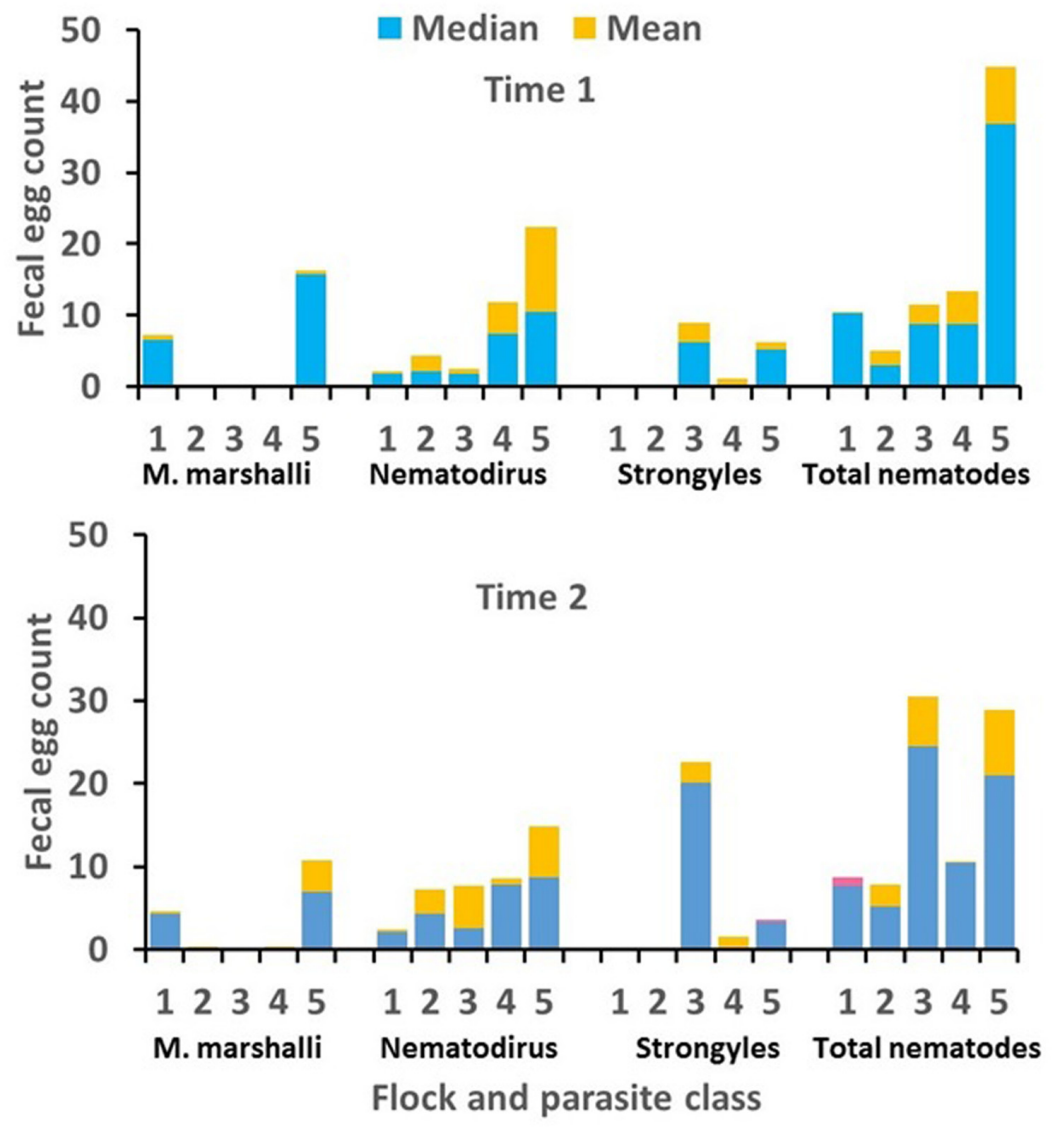
**Means and medians for Strongyle,* Nematodirus* sp., *Marshallagia marshalli*, and total nematode fecal egg counts (FEC; eggs/gram of feces) by flock and time of sampling.** Medians are generally shown by the height of the blue bars and means are shown by the total height of the blue plus orange bars. In rare cases where the median exceeded the mean, the median is shown by the combined height of blue plus pink bars and the mean is shown by the height of the blue bars.

Descriptive statistics for measured variables are shown in **Table 5**. The incidence of *Trichurus* infection in these data was very low. Eggs of *Trichurus* sp. were observed in fewer than 7% of the samples and FEC for Trichurus sp. were therefore excluded from the statistical analysis. Means and medians for FEC for remaining parasite classes are shown by flock and measurement time in **Figure 5**. Means for Strongyle FEC were very low in flocks 1 and 2, and means for *M. marshalli* FEC were very low in flocks 2, 3, and 4. The FEC records for these flocks for these parasite classes were therefore excluded from the final analyses.

**Table 5:** Descriptive statistics for fecal egg counts (FEC; eggs per gram of feces) for various classes of gastrointestinal nematodes and performance for 100 Ghezel lambs from five flocks.

Measurement	Mean	Median	SD	Minimum value	Maximum value
Strongyle FEC	4.44	0	9.38	0	61.25
*Nematodirus* FEC	8.41	3	13.55	0	73.50
*Trichurus* FEC	0.22	0	1.00	0	8.75
*Marshallagia* FEC	4.06	0	7.30	0	31.50
Total nematode FEC	171.28	105	202.24	0	997.50
Body weight, kg	31.84	31	5.89	19.7	47.50
PCV, %	33.04	33	3.93	21.0	46.00
FAMACHA score	1.78	2	0.74	1.0	4.00

Two fecal samples were obtained from each lamb. Samples were taken weekly beginning at approximately 31 days after deworming.

After removing FEC records for flocks 1 and 2 and expressing FEC as residual deviations from flock × time subclass means, the distribution of *M. marshalli* FEC was somewhat skewed to the right (ω = 0.49; *P* < 0.05) but did not exhibit significant kurtosis (κ = -0.04). This result was in agreement with the similarity between means and medians for *M. marshalli* FEC in **Figure 5**. A Box–Cox transformation with λ = 0.5 reduced the observed level of skewness (ω = -0.28), and a square-root transformation was used in the final analysis of *M. marshalli* FEC. As expected from differences between means and medians in **Figure 5**, *Nematodirus*, Strongyle, and total nematode FEC were not normally distributed. For these parasite classes, distributions of FEC were strongly skewed to the right (ω ≥ 1.28) and were leptokurtic (κ ≥ 3.51). Distributions of *Nematodirus* and Strongyle FEC also exhibited clumping at zero. Estimates of λ for Box–Cox transformations were close to zero for these parasite classes, with λ = 0.1 for total nematode and Strongyle FEC and λ = -0.1 for *Nematodirus* FEC. The Box–Cox transformation is undefined at λ = 0 but asymptotically approaches a logarithmic transformation as λ approaches zero. We therefore used a simple logarithmic transformation [ln(FEC + 1)] for these parasite classes.

**FIGURE 6:**
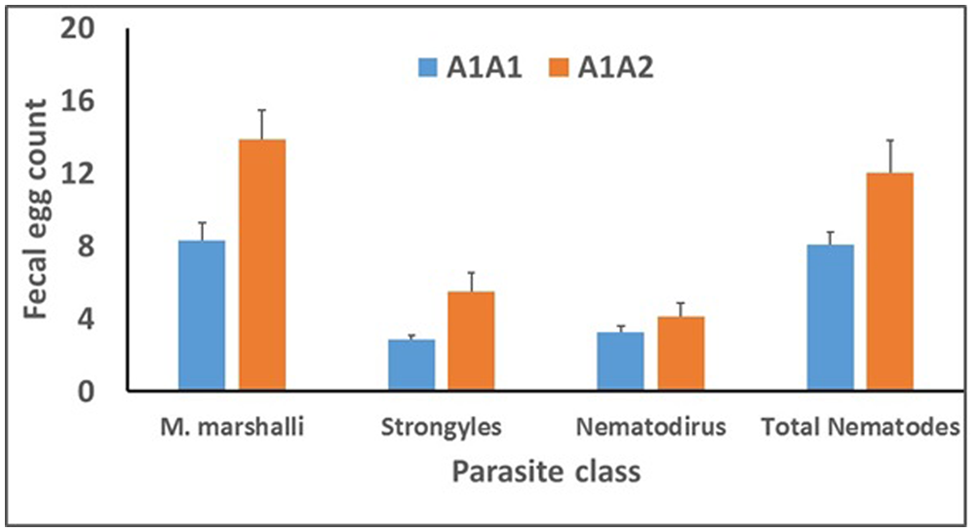
**Genotype means for FEC (egg/gram of feces) for the PstI restriction-fragment polymorphism in the Ovar-DRB1 gene for several parasite classes.** Values for Strongyle, *Nematodirus* sp., and total nematode FEC were transformed as ln(FEC + 1) for statistical analysis; least-squares means (m) were back-transformed to the original scale as (*e*^m^_-__1_) for presentation and are geometric, rather than arithmetic, means. Values for *M. marshalli* FEC were transformed as the square root of FEC for analysis; least-squares means shown here are based on untransfomed FEC.

Results of the repeated-measures analysis (**Table 6**) indicated that flock effects on FEC were large (*P* < 0.001) for all remaining parasite classes. Significant differnces were observed between Ovar_DRB1 genotypes A1A1 and A1A2 for *M. marshalli*, Strongyle, and total nematode FEC (**Figure 6**). Means for *M. marshalli* FEC in **Figure 6** were based on untransformed FEC and were 40% lower for lambs of genotype A1A1 compared to lambs of genotype A1A2. Means for Strongyle, *Nematodirus*, and total nematode FEC were backtransformed from means of transformed variables (*m*) as (*e*^m-1^), and SEs for backtransformed means were approximated by assuming that SEMs for log-transformed FEC were approximately equal to coefficients of variation of backtransformed means. Backtransformed means for these parasite classes indicated that Strongyle and total nematode FEC were 41 and 30% lower, respectively, for lambs of genotype A1A1 compared to lambs of genotype A1A2. No effect of genotype was observed for *Nematodirus* FEC Differences among genotypes were consistent among flocks and measurement times, with no effect of genotype × flock (*P* ≥ 0.23) or genotype × measurement time interaction (*P* ≥ 0.34 for any parasite class.

**Table 6:** Results of analysis of variance for fixed effects of flock, genotype, time of measurement, and their two-way interactions on FEC for each parasite class.

FEC for:	Effect	*F*-value	Pr > *F*
Total nematodes	Flock	20.13	< 0.001
	Genotype	4.42	0.04
	Time	0.56	0.46
	Genotype × flock	0.39	0.81
	Time × flock	4.31	< 0.01
	Genotype × time	0.02	0.90
Strongyles	Flock	17.98	< 0.001
	Genotype	6.24	0.02
	Time	1.68	0.20
	Genotype × flock	1.11	0.34
	Time × flock	12.14	< 0.001
	Genotype × time	0.93	0.34
*Marshallagia marshalli*	Flock	20.66	< 0.001
	Genotype	6.57	0.01
	Time	2.27	0.14
	Genotype × flock	1.48	0.23
	Time × flock	0.28	0.60
	Genotype × time	0.28	0.60
*Nematodirus*	Flock	6.16	< 0.001
	Genotype	0.73	0.39
	Time	1.55	0.22
	Genotype × flock	0.25	0.91
	Time × flock	1.29	0.28
	Genotype × time	0.01	0.93

Total nematode, Strongyle, and *Nematodirus* FEC were transformed as ln(FEC + 1) and *M. marshalli* FEC were transformed as the square root of FEC before analysis.

The impact of logarithmic transformation of Strongyle and total nematode FEC can be seen by comparing differences among genotypes for untransformed FEC. For untransformed FEC, means for lambs of genotype A1A1 were 29 (*P* = 0.20) and 23% (*P* = 0.12) lower compared to lambs of genotype A1A2. These differences in effect of genotype reflect the greater impact of occasional large FEC from the skewed right tail of the FEC distribution on mean differences between genotypes and on the residual variance. This issue could be addressed in untransformed data by removing records with very high FEC as outliers. We considered recoding of extreme values to be preferable to removing them, and use of a normalizing transformation provides an objective strategy to account for the presence of extreme values.

Results from analysis of untransformed FEC assuming a negative binomial distribution did not differ greatly from those from the analysis of transformed FEC. Means for untransformed *M. marshalli*, Strongyle, and total nematode FEC assuming a negative binomial distribution were 40 (*P* = 0.02), 18 (*P* = 0.01), and 43% (*P* = 0.15) lower, respectively, for lambs of genotype A1A1 compared to lambs of genotype A1A2. A significant effect of genotype was thus confirmed for *M. marshalli* and Strongyle FEC, but not for total nematode FEC, perhaps in association with different contributions of the various parasite classes to total nemadode FEC among flocks and measurement times.

Consistent effects of measurement time on FEC were not observed for any parasite class, but flock × measurement time interaction was significant for Strongyle and total nematode FEC (**Table 6**). The interaction was explained by a threefold increase in Strongyle FEC between the first and second measurement time in flock 3 (**Figure 5**). Significant difference in FEC between measurements times were not observed in other flocks.

**FIGURE 7:**
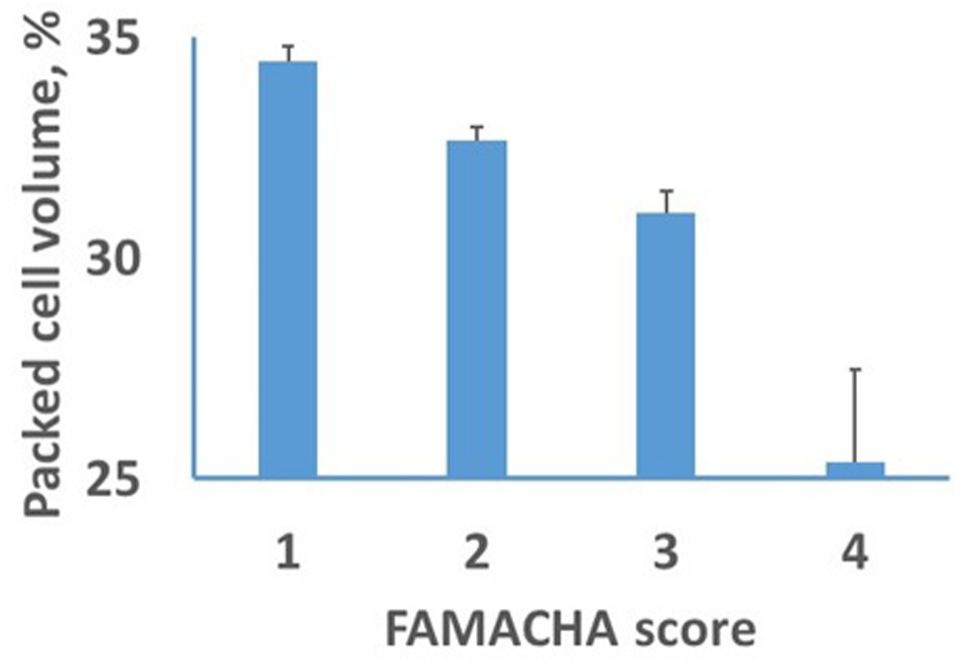
**Least-squares means and SEs for effects of FAMACHA score on packed cell volume (PCV)**.

Effects of genotype were not observed for lamb body weight or PCV. The distribution of FAMACHA scores revealed little evidence of anemia in these lambs, with frequencies across flocks and measurement times for FAMACHA scores of 1 through 5 of 40, 44,15, 1, and 0%, respectively. No association was observed beteween FAMACHA scores and lamb body weights or FEC for any parasite class. However, a significant association was observed between FAMACHA scores and lamb PCV (**Figure 7**). The PCV declined linearly as FAMACHA scores declined from 1 to 3 and were much lower for the two lambs that received a FAMACHA score of four. After adjusting for effects of herd and measurement time, a residual correlation of -0.51 (*P* < 0.001) was observed between PCV and the FAMACHA score.

## Discussion

Breeding for resistance to nematode infection can complement the use of anthelmintics in sheep husbandry. Resistant animals can be selected on the basis of low FEC (Eady et al., 2003; Kahn et al., 2003), and measurement of FEC is generally considered to be the standard method for assessment of the level of resistance to GIN. The number of eggs is easy to measure, indicates parasitism *per se*, and correlates well to the number of adult nematodes present in lambs (Douch et al., 1996; Baker, 1999). However, FEC is affected by several factors, such as parasite fecundity and egg-laying patterns, variations in egg distribution in feces, diet composition, intestinal transit time, and the level of immunity (Douch et al., 1996). A disadvantage of FEC as a marker of resistance is the requirement that animals be infected, either naturally or artificially, to determine the FEC value. The effort and cost in obtaining FEC measurements can also be a disadvantage, especially under extensive production conditions. Under some production conditions, it is therefore difficult to assess the resistance status of animals for breeding programs and there is, consequently, considerable interest in the evaluation of phenotypic and genetic markers associated with parasite resistance. Other phenotypic measures such as degree of anemia, circulating eosinophil counts, antibody levels to larval, or adult stages, and plasma pepsinogen concentrations can be used to predict worm burdens and resistance levels in infected sheep, but phenotypic markers that allow accurate prediction of an individual’s resistance status in the absence of infection are generally not available (Albers et al., 1987; Beh and Maddox, 1996).

A number of studies around the world have attempted to identify relationships between genetic resistance to GIN and various genes and genetic markers. Some of these studies have been summarized in **Table 7**. Polymorphisms within the ovine MHC complex were associated with resistance to *T. colubriformis* (Douch and Outteridge, 1989), *T. circumcincta* (Schwaiger et al., 1995), and *H. contortus* (Luffau et al., 1990; Outteridge et al., 1996). However, Cooper et al. (1989), Blattman et al. (1993) and Hulme et al. (1993) did not find evidence of an association between polymorphisms in the ovine MHC locus and resistance or susceptibility to *H. contortus*. A list of QTL for GIN resistance in sheep was provided by Dominik (2005). This review suggests that there is considerable evidence for an important role for the MHC in parasite susceptibility and resistance to *H. contortus*.

**Table 7:** Comparision of of results of the current study with other investigations of Ovar-DRB1 or nearby genes.

Breed	Country	Method	Gene	Association	Reference
Suffolk	Ireland	PCR products sequenced	Ovar-DRB1	Low FEC	Sayers et al. (2005)
Romanov	Poland	Microsatellite	Ovar-DRB1	Low FEC	Charon et al. (2002)
Scotish Blackface	Scotland	Simple Tandom Repeat	Ovar-DRB1	Low FEC	Schwaiger et al. (1995)
Pelibuey	Mexico	Microsatellite	Ovar-DRB1	LowFEC	Figueroa Castillo et al. (2011)
Corriedale	Brazil	Microsatellite	IL- 4	Low FEC	Benavides et al. (2009)
Texel	Ireland	Microsatellite	IFN-γ	Low FEC	Sayers et al. (2005)
Soay	Scotland	Microsatellite	IFN-γ	Low FEC	Coltman et al. (2001)
**Ghezel**	**Iran**	**PCR-RFLP**	**Ovar-DRB1**	**Low FEC**	**Current study**

Sallé et al. (2012) reported four QTL regions on sheep chromosomes (OAR) 5, 12, 13, and 21 in Romane X Martinik Black Belly backcross lamb that had an important role in genetic resistance to *H. contortus*. Riggio et al. (2014) suggested other regions of OAR1, 3, 4, 5, 7, 19, 20, and 24 that were involved in GIN resistance. Davies et al. (2006) likewise found evidences for QTL on chromosomes 2, 3, 14, and 20 that were associated with parasitic infections in Scottish blackface sheep.

Results of the current study provided additional evidence of an association between polymorphism in the DRB1 gene and GIN FEC, and the first indication of an effect of this locus on *M. marshalli* FEC. Screening of GIN FEC under natural infection is informative because it corresponds to typical conditions in the production environment. However, possible interactions among effects of different parasite classes and variation among flocks and measurement times in parasite loads preclude a deeper understanding of mechanisms driving the observed associations. More intersive studies, involving controlled infections with individual species of GIN are thus required to confirm hypothesized effects of DRB1 genotype on GIN parasite resistance and to confirm the specificity or generality of observed associations among parasite classes.

A significant negative association between PCV and FAMACHA score confirmed that the FAMACHA score can be used to diagnose differences in PCV in lambs and was consistent with previous results (Kaplan et al., 2004). Recommendations for veterinary intervention based on FAMACHA scores (Vatta et al., 2001) suggest that lambs with scores of one or two do not require attention, but that lambs with scores of four or five require immediate attention. Recommendations for animals with a score of three depend upon the age and nutritional status of the lamb and anticipated cause(s) of anemia. Intervention is generally recommended for lambs, but not adults, with a FAMACHA score of three. In the current study, the lack of association between FAMACHA scores and FEC, low overall FEC levels, and limited evidence for parasitism by *H. contortus* (the only GIN, of those evaluated, that causes blood loss and anemia) suggest that parasitism probably is not the main cause of subclinical anemia in these flocks. Nonetheless, results in **Figure 7** indicate that FAMACHA scores can be used to detect mean differences in PCV among lambs.

## Conclusion

Our results reinforce previous evidence that some alleles of the ovine MHC are involved in determining levels of susceptibility or resistance to infection with GIN. This result provides the opportunity to use these alleles as genetic markers of resistance to GIN, leading to the development of that are better adapted to parasite infestations in the environment.

Implication of this research are that FAMACHA tests and polymorphic markers of Ovar-DRB1 can be used in applied animal breeding programs on sheep farms of the region, especially in animals infected with GIN and located in the temperate regions of Asia. Assessment of the precision of genetic evaluations based on molecular information has potential to provide a new perspective on the design of sheep breeding schemes and selection programs (Assenza et al., 2014).

## Conflict of Interest Statement

The authors declare that the research was conducted in the absence of any commercial or financial relationships that could be construed as a potential conflict of interest.
